# T-cell activation discriminates subclasses of symptomatic primary humoral immunodeficiency diseases in adults

**DOI:** 10.1186/1471-2172-15-13

**Published:** 2014-03-12

**Authors:** Marie-Quitterie Picat, Rodolphe Thiébaut, François Lifermann, Xavier Delbrel, Daniel Adoue, Linda Wittkop, Anne-Laure Fauchais, Patrick Rispal, Jean-François Moreau, Jean-François Viallard

**Affiliations:** 1 INSERM, ISPED, Centre INSERM U897-Epidemiologie-Biostatistique, Bordeaux F-33076, France; 2 University of Bordeaux, ISPED, Centre INSERM U897-Epidemiologie-Biostatistique, Bordeaux F-33076, France; 3 Department of Medical Information, Bordeaux University Hospital, Bordeaux F-33000, France; 4 INRIA SISTM, F-33405 Talence, France; 5 Department of Internal Medicine, Dax Hospital, Dax F-40107, France; 6 Department of Internal Medicine, Pau Hospital, Pau F-64046, France; 7 Department of Internal Medicine, Purpan Hospital, Toulouse F-31059, France; 8 Department of Internal Medicine, Dupuytren Hospital, Limoges F-87042, France; 9 Department of Internal Medicine, Agen Hospital, Agen F-47923, France; 10 University of Bordeaux, Bordeaux F-33076, France; 11 CNRS, UMR 5164, Bordeaux F-33076, France; 12 Bordeaux University Hospital, Laboratory of Immunology, Bordeaux F-33076, France; 13 Department of Internal Medicine and Infectious Diseases, Bordeaux University Hospital, Pessac F-33604, France; 14 Hôpital Haut-Lévêque - Service de médecine interne, 5, avenue de Magellan, Pessac 33604, France

**Keywords:** Symptomatic primary humoral immunodeficiency, T-cell activation, HLA-DR marker, Hierarchical clustering, Principal component analysis, Common variable immunodeficiency, IgG subclass deficiency, Good’s syndrome

## Abstract

**Background:**

Symptomatic Primary Humoral Immunodeficiency Diseases (PHID) constitute a highly heterogeneous group of diseases characterized by a shared hypogammaglobulinemia, resulting in increased risk of recurrent or severe infections. Associations have been described with a variety of immunological abnormalities involving B and T-cell differentiation, T-cell activation and innate immunity. However, PHID discrimination remains based on B-lymphocyte abnormalities and other components of the immune system have not been sufficiently taken into account. We carried out unsupervised and supervised methods for classification in a cohort of 81 symptomatic PHID patients to evaluate the relative importance of 23 immunological parameters and to select relevant markers that may be useful for diagnosis and prognosis.

**Results:**

We identified five groups of patients, among which the percentage of PHID complications varied substantially. Combining the set of markers involved in PHID supported the existence of two distinct mechanisms associated with complications. Switched memory B-cell attrition and CD8+ HLA-DR + activated T-cell increase were the prominent abnormalities observed in PHID complications. Furthermore, in a subgroup of 57 patients with common variable immunodeficiency, the classification that added CD8+ HLA-DR + to the consensual EUROclass classification was better than the EUROclass model in predicting complications.

**Conclusion:**

These results highlight the importance of T-cell activation that may improve discrimination of PHID patients in specific subgroups and help to identify patients with different clinical outcomes.

## Background

Primary Humoral Immunodeficiency Diseases (PHID) are a heterogeneous group of diseases characterized by a deficit in immunoglobulin (Ig) production, resulting in increased risk of recurrent or severe infections [1]. In adults, after elimination of secondary causes, in particular lymphoid hemopathies, drug-related causes or renal/digestive leakage of Ig, three major symptomatic PHID have to be entertained: Common Variable ImmunoDeficiency [2] (CVID), IgG SubClass ImmunoDeficiency [3] (IgG SD) or Good’s syndrome [4]. Although recurrent respiratory tract infections is common to nearly all patients with symptomatic PHID, distinct clinical complications have been described [5-7] according to the occurrence of autoimmune manifestations, lymphoid hyperplasia, chronic enteropathy, splenomegaly and/or granulomatous disease. It has been shown that both outcome and prognosis vary within these subgroups of phenotypes in PHID [4,6]. Immunoglobulin substitution reduces the incidence of acute infections but does not solve the appearance of complications that are the major cause of morbidity and death among patients with symptomatic PHID [4,8].

The various attempts of PHID classifications have focused on CVID because of their frequency [2]. Different classification proposals have attempted to define CVID using flow cytometry techniques based on B-cell differential phenotyping [9,10]. Recently, data from the EUROclass group including over 300 European CVID patients, unified previous findings with a classification based on the percentage of B cells among circulating lymphocytes and the percentage of switched memory B cells (smB) among B cells [5]. However, this updated classification does not include other components of immune dysregulation, either causal or consequential, that have been reported in CVID patients. Giovanetti et al. [11] performed an in-depth analysis of the T-cell compartment in CVID patients and demonstrated multiple T-cell abnormalities. T-cell activation is an important process underlined [12,13] that need to be explored, together with T-cell regulation [14,15]. Indeed, gammadelta T cells [16] and innate cell abnormalities reported in Natural Killer cells (NK) [17] or myeloid dendritic (mDC) and plasmacytoid dendritic cells (pDC) [18] may further contribute to the heterogeneous presentation of CVID. Literature on IgG subclass deficiency is poor and immunological characteristics of these patients have not been explored as they have been in CVID. In parallel, Good’s syndrome is characterized by hypogammaglobulinemia and thymoma, low or absent B cells, variable defects in cell-mediated immunity with a CD4 T lymphopenia, an inverted CD4/CD8+ T-cell ratio and reduced T-cell mitogen proliferative responses [4].

Because of its intrinsic complexity, distinguishing patients with symptomatic PHID in practice can be challenging. Particularly, the median delay of diagnosis reported in CVID is 7 years [2]. Some patients with IgG subclass deficiency might develop non infectious complications as those observed in CVID [19], suggesting that these two entities could have common determinants. Moreover, IgG subclass deficiency may progress to have typical common variable immunodeficiency [3]. The immunophenotypic evaluation of symptomatic PHID could provide diagnostic clues as well as information useful to manage patients, to predict clinical outcomes [20,21] and to improve classification schemes. The purpose of our study was therefore to characterize subgroups of PHID patients that could be defined by differentiation, activation and regulation markers of B and T-lymphocytes expressing αβ or γδ TCR, natural killer cells and dendritic cells using supervised and unsupervised methods of classification appropriate for the high-dimensional data [22] generated by flow cytometry [23,24].

## Methods

### Design and data collection

The ALTADIH Cohort is a prospective hospital-based cohort of symptomatic PHID patients [1], initiated at the Bordeaux University Hospital and five other public hospitals (Agen, Dax, Limoges, Pau and Toulouse) in Aquitaine, South-Western France. First patients were enrolled in 2007. Patients included were over eighteen years old with primary hypogammaglobulinemia (serum IgG level < 5 g/L for CVID, or IgG subclass deficiency or Good’s syndrome). They were already followed for symptomatic or newly diagnosed PHID (Ig level determined before substitution therapy). Differential diagnoses of hypogammaglobulinemia have been excluded and the reduction in the level of the seric gammaglobulins was confirmed at least 3 times at 3-month intervals for each patient, thus excluding transient hypogammaglobulinemia. A standardized questionnaire was filled by physicians at each hospital contact, according to routine clinical management procedures, generally every 6 months. Clinical data collected focused on infections and PHID complications while biological data focused on innate and adaptive markers.

The ALTADIH Cohort was approved by the Bordeaux University Institutional Review Board on December 20th, 2006. Each patient gave informed written consent before participating in the study. Some PHID patients were also enrolled in the French national prospective Cohort DEFI [7].

In this paper, the study is cross-sectional. We only deal with inclusion measurements.

### Flow cytometric analysis of peripheral blood lymphocytes

Peripheral blood samples were collected at each visit for systematic lymphocyte phenotyping of PHID patients under or not current immunoglobulin substitution. Blood samples from each contributing center were analyzed centrally at the Bordeaux University Hospital Laboratory of Immunology, by flow cytometry using a FC500 flow cytometer from Beckman-Coulter.

All blood samples were withdrawn on EDTA anti-coagulant in Vacutainers 5 ml tubes (BD, Biosciences, Mountain View, CA) and kept at room temperature until processed. All samples were analyzed fresh following withdrawal within a working day. Following manufacturer recommendations, labeling were carried out on whole blood and red blood cells lyzed at room temperature with a Versalyse (Beckman-Coulter, France, ref = A0937) lysing solution added with Iotest fixative solution (Beckman-Coulter, France, ref = A07800). Events were acquired with the dedicated CXP-1 software. In these settings, we did not use any viability staining.

Unless specified, all monoclonal antibodies were purchased from Beckman-Coulter (France) whose catalog references are reported below and in Additional file 1: Table S1. We used the following panel of monoclonal antibodies (Mab):

– B cells subsets: anti- CD19 (ref: A07766), -CD27 (ref: 6607107), -CD21 (ref: PN IMU473U) and -sIgD (ref: 736000)

– CD8+ T-cell differentiation: anti-CD8 (ref: A07756), -CD45RA (ref: PN IM 271U), -CCR7 from R&D (Minneapolis, MN, USA ref: FAB197A)

– HLA-DR T-lymphocyte activation markers, we used the following panel of Mab: anti-CD45, -3, -4 and -8 (ref: 6607013) -HLA-DR (ref: PNA40579).

– CD25 T-lymphocyte activation marker: anti-CD3, -4 and -8 (ref: 6607013) -CD25 (ref: IM2646)

– CD28 T-lymphocyte activation marker: anti-CD3, -4 and -8 (ref: 6607013) -CD28 (ref: 6607108)

– CD38 T-lymphocyte activation marker: anti-CD3, -4 and -8 (ref: 6607013) -CD38 (ref: A07780)

– regulatory T-cell: anti-CD4 (ref: A07750), -CD25 (ref: IM2646) -CD127 (ref: PN IM1980U)

– NK and B cells: anti-CD45, -56, -19 and -3 (ref: 6607073) -CD16 (ref: A07766)

myeloid and plasmacytoid dendritic cells, we used the Beckman Coulter kit (ref: A23413 for mDC and A23416 for pDC).

– T cells expressing the gamma/delta TCR: anti-Vdelta2 (ref: PN IM1464) and anti-pan-delta (ref: PN IM1418U).

T and B-cell subpopulation counts were obtained using the flow count beads kit from Beckman-Coulter Flow Count (ref: 7547053) following a lyse and no wash procedure according to the manufacturer's recommendations. Markers of CD4+ T-cell differentiation were not performed in this study.

Results are expressed as percentages of total circulating lymphocytes or of CD19+, CD3+, CD4+ or CD8+ lymphocytes as appropriate, and/or absolute counts in cells/mm3 as appropriate. An exception are dendritic cells, whose numbers are expressed per milliliter of blood, obtained by calculations from the leukocyte count.

Concerning the gating strategies, as examples, we choose to show in Additional file 2: Figure S1, the gating strategy used to assessed CD3+, CD3 + CD4+, CD3 + CD8+, and the proportion of cells expressing HLA-DR among these subsets: in Panel A, applied to a normal healthy donor whereas, in Panel B, applied to a CVID patient. Panel C depicts the B/NK cells phenotyping of the CVID patient.

### Statistical analyses

Analyses were performed using SAS® 9.1 (SAS Institute, Inc., Cary, NC) and XLSTAT® 2010 software (Addinsoft, Paris, France). Clinical and therapeutic data were described as numbers and percentages. Immunoglobulin serotypes and immunological markers were described as medians and InterQuartile Range values (IQR). We conducted multidimensional exploratory analyses (principal component analysis and cluster analysis) to study the links between immunological markers and clinical characteristics. To run the multidimensional exploratory analyses, CD19+ B cells, CD3+, CD4+, CD8+ T cells, regulatory T cells, NK cells and gamma/delta T-cells have been used in absolute counts (cells/mm^3^). CD3 + HLA-DR + activated T cells have been used in percentages of CD3+, CD4 + HLA-DR + in percentages of CD4+, and CD8 + HLA-DR + in percentages of CD8+. Differentiation markers of T-cells and CD8 + CD57+ have been used in percentages of CD8+ and differentiation markers of B-cells have been used in percentages of CD19+. Numbers of dendritic cells were expressed per milliliter of blood and gamma/delta2 T-cells in percentages of gamma/delta T-cells. The use as absolute numbers or percentage has been defined before performing any statistical analyses (see Tables with each immunological marker for a summary of the units used to run the multidimensional exploratory analyses). Robustness analyses have been performed using absolute count in place of percentage and vice versa and results were not substantially different.

#### Principal component analysis

**Figure 1:**
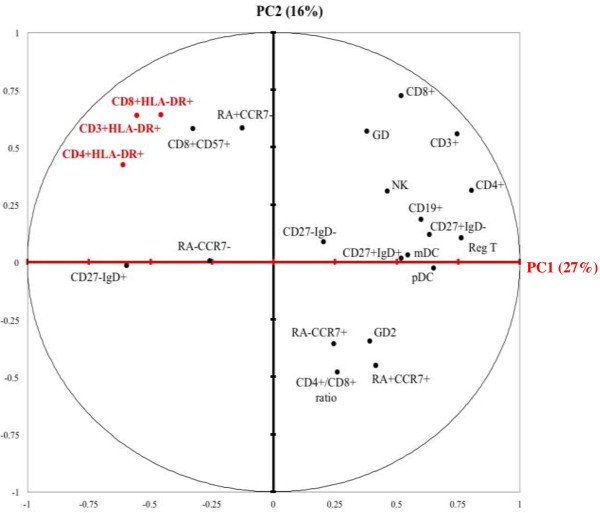
**23 immunological markers involved in PHID, plotted on the first two Principal Components (PC).** ALTADIH Cohort, 2007-2010. Immunological markers are well represented by the component when they are far from the center and close to the corresponding axis. Two markers are: 1) positively correlated if close to each other; 2) not correlated if in a rectangular position; 3) negatively correlated if on the opposite side.

Principal Component Analysis (PCA) is an unsupervised dimension-reduction method that generates Principal Components (PC), that are linear combinations of the original variables [25] (in our case, represented by differentiation, activation and regulation markers of B and T-lymphocytes, natural killer cells, dendritic cells and T-cells expressing a gamma/delta TCR). Each component explains a part of the variability of the data. We used 2D plots to project data on the plane spanned by the first two components to display 23 immunological markers involved in PHID (missing data excluded). As data run in the principal component analysis have been normalized as referred to the sample (minus sample mean and divided by standard deviation), data are presented on a unit correlation circle of radius 1 (see Figure 1). Because each component is a weighted linear combination of the original variables, a component may have a meaning according to the contribution of each immunological marker. We chose to present the plan spanned by the two major principal components because they explained the most, variability of data (> 10%). On this plan, the contribution of markers is unequal and depends on the contribution of each marker on each axis. A given marker may contribute on the third axis and therefore would not be well represented on the first two axes. PCA was also performed in CVID patients. A PCA on markers of 12 healthy donors is given in Additional file 3: Figure S2.

#### Cluster analysis

Hierarchical clustering is a common method used to determine clusters of similar data points [26]. Ward’s hierarchical clustering method was used here [27]. This method aggregates consecutively the patients presenting the closest immunological characteristics, by maximizing inter-class variance using Ward’s criterion. This process leads to the construction of a classification tree (patients with missing immunological data excluded), which allowed us to identify some distinct subgroups of PHID patients. The projection of the identified clusters onto the first two principal components then helped us characterize them according to immunological markers. A cluster analysis was also performed in CVID patients.

#### PHID complication analyses

PHID complications were described in each subgroup of patients identified by the cluster analysis in order to provide clinical-immunological links. These complications are those defined by H Chapel et al. [6] to distinguish different clinical phenotypes (autoimmunity, lymphoid hyperplasia and chronic enteropathy) and granulomatous disease and splenomegaly in addition [12]. Univariable logistic regression was performed to study the association between PHID complications and the 23 immunological markers (variables with a p-value < 0.25 were further tested). A forward-selection procedure was used to build a final multivariable model. The first marker included in the model was the most significant in univariable analysis. The next variable included was the one with most significant association with complications in the remaining set and so on until any additional marker became non significant. As the immunological markers in our study were expressed in different units, we reported the Odds Ratio (OR) per 1 standard deviation for a unit free representation.

### CVID substudy

The most consensual classification in symptomatic PHID concerns CVID. EUROclass [5] is the classification commonly used in CVID, segregating patients with nearly absent B cells (less than 1%), severely reduced switched memory B cells (less than 2%), and expansion of CD21^low^ B cells (more than 10%). To evaluate if T-cell markers could improve this classification [28], we evaluated the improvement in predictive value for CVID complications by a net reclassification approach [29]. To do so, we estimated predicted probabilities of CVID complications using two models: one with EUROclass, the other with EUROclass and CD8+ HLA-DR + (the most significantly associated in our analyses). Based on these two models, we categorized the predicted probabilities into two clinical meaningful categories (probability < 50% or ≥ 50%) and cross-tabulated the classifications. The improvement in reclassification can be quantified as the sum of differences in proportion of individuals moving up minus the proportion moving down for people who developed complications and the proportion of individuals moving down minus the proportion moving up for people who didn’t develop complications. Predictive capacity was also evaluated using the area on the receiving operating characteristics curve (c statistics).

## Results

### Patient characteristics

#### Sociodemographic and clinical characteristics

Between January 2007 and March 2010, 81 PHID patients were enrolled in the ALTADIH Cohort (among whom 33 were also included in the DEFI Cohort), including 57 CVID patients, 21 IgG subclass deficit patients and 3 patients with Good’s syndrome. At the time of evaluation, none of the PHID patients had evidence of an acute infection. The majority of patients were followed at the Bordeaux University Hospital (66 patients). Baseline epidemiological and clinical characteristics are reported in Table 1. Of the 81 patients, 59% were women. The median age at PHID diagnosis was 41 years old (IQR: 35–54). The most frequent symptoms in disease histories were respiratory tract infections. Altogether, 38% of the patients developed one or more complications: splenomegaly, autoimmune manifestations, granulomatous disease, lymphoid hyperplasia and/or chronic enteropathy (Table 1). More than 80% of the patients were given subcutaneous or intravenous immunoglobulin substitution regimen.

**Table 1:** Baseline characteristics of 81 PHID patients, ALTADIH Cohort, 2007-2010

**Characteristics**	**Distribution**	
**Sociodemographic data**		
Female, n (%)	48	(59)
Median age at diagnosis in years (interquartile range)	41 (35–54)	
Median age at enrolment in years (interquartile range)	46 (38–58)	
**Clinical data, n (%)**		
**Respiratory tract infections**		
Otitis, sinusitis, nasopharyngitis, polyposis	64	(79)
Bronchitis, pneumonia	65	(80)
Bronchiectasis	21	(26)
**Gastrointestinal tract infection**		
Chronic diarrhea	16	(20)
Acute diarrhea	13	(16)
Lambliasis	11	(14)
**PHID complications: at least one of the following**	31	(38)
Lymphoid hyperplasia	16	(20)
Splenomegaly	14	(17)
Autoimmune manifestations*	14	(17)
Granulomatous disease^†^	9	(11)
Villous atrophy	5	(6)
Chronic inflammatory intestinal disease	4	(5)
**Therapeutic data, n (%)**		
Subcutaneous or intravenous immunoglobulin substitution	66	(81)

*Autoimmune anemia, autoimmune neutropenia, autoimmune thrombocytopenia, Hashimoto thyroiditis, Goujerot-Sjögren syndrome.

^†^Location of biopsy proven granuloma: lymph node, digestive tract, spleen, bone narrow.

#### Biological characteristics

Immunoglobulin serum levels before substitution for IgG, IgA and IgM in PHID patients were significantly reduced: baseline levels IgG (median: 4.3, IQR: 3.1–5.3 g/L; normal range: 6.6–12.8 g/L), IgA (median: 0.5, IQR: 0.05–1.2 g/L; normal range: 0.7–3.4 g/L), IgM (median: 0.4, IQR: 0.2–0.8 g/L; normal range: 0.5–2.1 g/L). Specifically, in CVID patients, median baseline IgG was 3.8 g/L (IQR: 1.7-4.4 g/L), median baseline IgA was 0.3 g/L (IQR: 0.05-1.00 g/l) and median baseline IgM was 0.3 g/L (IQR: 0.1-0.6 g/L). Baseline immunological marker values in PHID patients are reported in Table 2 (values of normal patients are reported in Additional file 4: Table S2). Some patients were included in ALTADIH at diagnosis, before the introduction of IgG replacement therapy, and had a longitudinal follow-up of their immunological parameters. The replacement therapy did not change the values of these parameters on two years of monitoring.

**Table 2:** Baseline immunological marker values of 81 PHID patients, ALTADIH Cohort 2007-2010

		**Unit**	**Median**	**IQR**
**Total circulating lymphocytes (TCL)**		cells/mm^3^	1384	(1017, 2000)
**B cells**	CD19+	cells/mm^3^	128	(62, 193)
		% TCL	9.37	(6.21, 12.91)
Naïve	CD27-IgD+	% CD19+	74.01	(56.42, 83.35)
Switched	CD27-IgD-	% CD19+	2.62	(1.86, 5.16)
Marginal zone	CD27 **+** IgD+	% CD19+	9.42	(4.70, 15.37)
Switched memory	CD27 **+** IgD-	% CD19+	8.07	(3.63, 19.45)
**T cells**	CD3+	cells/mm^3^	1078	(746, 1585)
	CD4+	cells/mm^3^	657	(422, 941)
	CD8+	cells/mm^3^	329	(228, 524)
	CD4+/CD8+ ratio		1.99	(1.30, 2.70)
***CD8+ T cells***				
Naïve	CD45RA + CCR7+	% CD8+	18.01	(6.79, 36.07)
Central memory	CD45RA-CCR7+	% CD8+	1.25	(0.59, 2.48)
Effector memory	CD45RA-CCR7-	% CD8+	34.39	(24.47, 46.90)
Terminal effector	CD45RA + CCR7-	% CD8+	33.48	(23.31, 48.26)
Immunosenescent	CD8 + CD57+	% CD8+	15.97	(8.04, 31.75)
***Activated T cells***				
HLA-DR	CD3 + HLA-DR+	% CD3+	12.87	(7.99, 20.37)
	CD4 + HLA-DR+	% CD4+	7.96	(5.14, 13.79)
	CD8 + HLA-DR+	% CD8+	24.49	(14.31, 41.11)
**Regulatory T cells**	CD4 + CD25 + CD127-	cells/mm^3^% TCL	32 1.10	(16, 63) (0.51, 1.76)
**Natural Killer cells**	CD3-CD16 + CD56+	cells/mm^3^	126	(75, 194)
**Dendritic cells**				
Myeloid	mDC	/ml	9381	(6597, 15611)
Plasmacytoid	pDC	/ml	4671	(2810, 7427)
**Gammadelta cells**		cells/mm^3^	33	(15, 72)
Gammadelta 2 cells		% GD	56.32	(22.19, 77.17)

IQR, interquartile range.

### Principal component analysis of differentiation, activation and regulation markers of B and T-lymphocytes, natural killer cells, dendritic cells and T-cells expressing a gamma/delta TCR

For studying the association between the various immunological markers reported in the pathogenesis of PHID, statistical analyses would have been hampered by the high number of markers studied, some of them being highly correlated (e.g. activation markers). In order to circumvent these biases, we used PCA to reduce the multidimensionality of the dataset and capture immunologically relevant information. The first two principal components accounted for 43% of the variability among immunological markers (Figure 1). The first principal component, accounting for 27% of the marker variability, separated regulatory T-cells from activated cells (HLA-DR + on CD3+, CD4+ and CD8+ grouped together) and CD19 + CD27 + IgD- switched memory B cells from CD19 + CD27-IgD + naïve B cells. The second principal component, which accounted for 16% of variability, mainly represented T-cell differentiation, opposing CD45RA + CCR7+ naïve CD8+ T cells and CD45RA + CCR7- terminal effector CD8+ T cells. Activated CD3+, CD4+ and CD8+ T cells expressing HLA-DR + were positively correlated to each other in PCA (Spearman rank correlation coefficient r = 0.88 between CD3+ HLA-DR + and CD4+ HLA-DR+; r = 0.90 between CD3+ HLA-DR + and CD8+ HLA-DR+; r = 0.70 between CD4+ HLA-DR + and CD8+ HLA-DR+). HLA-DR + activation markers were also correlated to CD45RA + CCR7- terminal effector CD8+ T cells and to CD8 + CD57+, known to be a marker of senescence [30]. Hence, this first analysis and an additional analysis extended to 38 markers available on 72 patients including CD21+ B cells (see Additional file 5: Figure S3) led us to conclude that all activation markers were highly correlated and one of them could be chosen to reflect alone the T-cell activation process. In addition, the first two components defined an upper-left corner grouping HLA-DR + activation markers with CD45RA + CCR7- terminal effector CD8+ T cells and the CD8 + CD57+ senescence marker.

PCA using 23 immunological markers of CVID patients only (excluding IgG SubClass Immuno deficient and Good`s patients) gave consistent results as those observed with the whole PHID population.

### Classification of PHID patients according to cluster analysis

**Figure 2:**
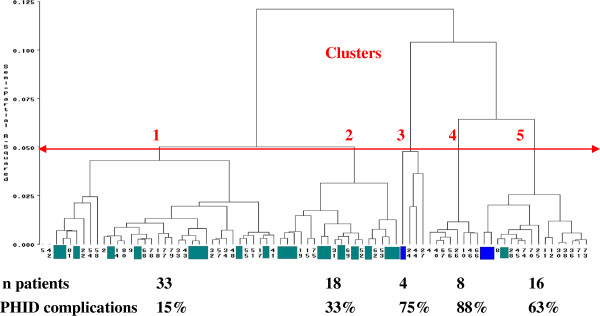
**Classification of 79 PHID patients by hierarchical cluster analysis, according to 23 immunological markers.** ALTADIH Cohort, 2007-2010. The arrow defines the number of clusters. Patients with IgG subclass immunodeficiency are depicted in green. Patients with Good’s syndrome are depicted in blue. Other patients are CVID patients. The percentage of PHID complications by cluster is indicated.

Next, an unsupervised classification was performed to define groups of patients that would be more homogeneous according to immunological markers. Hierarchical cluster analysis could be performed for 79 PHID patients of the 81 in the ALTADIH cohort (2 CVID patients not classified). Based on cubic clustering criterion, we would have chosen two clusters but the identification of five populations of PHID patients, as revealed by the five branches of the tree down stream (Figure 2), was chosen as these five clusters were clinically relevant. Clusters 1 and 2, made up by patients with CVID or IgG subclass immunodeficiency, were grouped together, separated from clusters 3, 4 and 5, mainly made up by patients with CVID or either Good’s syndrome. In the cluster analysis in CVID diagnoses (excluding patients with Good’s syndrome and IgG subclass immunodeficiency), the patients were grouped in the same way than in the previous cluster analysis performed on the whole population (identification numbers of CVID patients given for detailed parallel between Figure 2 and Additional file 6: Figure S4).

**Figure 3:**
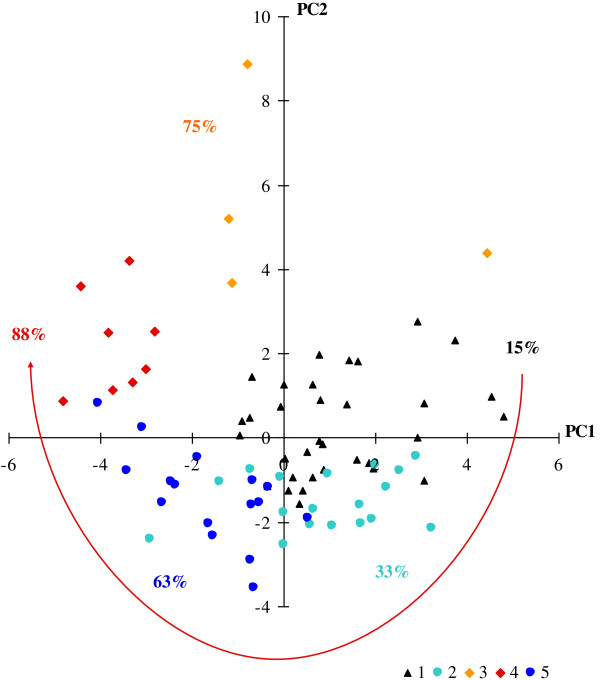
**Immunological interpretation of 5 clusters of CVID plotted on the first two principal components.** ALTADIH Cohort, 2007-2010. The percentage of CVID complications, noted next to the clusters, increased in a clockwise manner as phenotypes approach the projection of activated HLA-DR + markers plotted in Figure 1 (red arrow).

The projection of PHID patients, classified in the above 5 defined clusters, onto the plan spanned by the first two principal components led to define their immunological characteristics (Figure 3 that needs to be read in conjunction with Figure 1; Table 3). For example, patients plotted in the upper-left corner of Figure 3, mainly patients of cluster 4, are characterized by variables plotted in the same quadrant in Figure 1, in particular HLA-DR + markers. Thus, the 33 patients making up cluster 1 (23 CVID patients and 10 IgG SD patients) were characterized by normal innate and adaptive immunity. They were represented on the upper-right corner that grouped innate cells, CD19+ B-cells, CD19 + CD27 + IgD- switched memory B-cells, CD3+, CD4+, CD8+ T-cells, and regulatory T-cells (Figure 1). Cluster 2 (lower-right corner, Figure 3) represented 18 patients (8 CVID patients and 10 IgG SD patients) characterized by increased naïve CD8+ T-cells. Cluster 1 and cluster 2 looked similar and were combined in an up stream clustering. Cluster 3 (1 patient with Good’s syndrome and 3 CVID patients) and cluster 4 (8 CVID patients) were characterized by an increase of HLA-DR + activation, terminal effector CD8+ T cells and immunosenescence marker (Table 3). As expected, these patients were represented in the upper-left corner near CD3+, CD4+ and CD8+ T cells expressing HLA-DR+, CD45RA + CCR7- terminal effector CD8+ T cells, and the CD8 + CD57+ marker (Figure 1). In cluster 3, one CVID patient had been splenectomized before enrolment (in 2001). In this patient, splenectomy deeply affects the absolute numbers but not grossly the percentages of the lymphocyte subpopulations in the blood. In parallel, the patient with Good’s syndrome in cluster 3 was differently plotted, i.e. in the upper-right corner near CD8+, reflecting the high blood CD8+ T-cell absolute numbers known in Good’s syndrome. Cluster 5 (lower-left corner, Figure 3) consisted of 16 patients with B-cell deficiency (13 CVID, 1 IgG SD and 2 Good’s syndrome): the lower-left corner where no immunological parameters are plotted has to be interpreted as the symmetrical and diagonal opposite of the upper-right corner. Thus, the meaning of cluster 5 (lower-left corner, Figure 3) is a cluster gathering B-cell deficiencies because the patients are plotted on the opposite corner of the plot of CD19+, CD27 + IgD+, CD27 + IgD-, CD27-IgD- which all are markers plotted in the upper-right corner. Table 3 confirms the given interpretation of the clusters.

**Table 3:** Baseline immunological characteristics of 5 clusters of 79 PHID patients, ALTADIH Cohort, 2007-2010

		**Cluster 1**	**Cluster 2**	**Cluster 3**	**Cluster 4**	**Cluster 5**
		**n = 33**	**n = 18**	**n = 4**	**n = 8**	**n = 16**
IgG	g/L	4.37 (3.80, 5.32)	5.73 (4.7, 6.3)	4.68 (2.69, 5.29)	1.44 (0.30, 2.71)	3.69 (1.65, 4.22)
IgA	g/L	0.63 (0.14, 1.62)	1.02 (0.07, 1.55)	0.60 (0.04, 2.09)	0.15 (0.01, 0.43)	0.19 (0.05, 0.79)
IgM	g/L	0.55 (0.31, 1.09)	0.48 (0.24, 0.78)	0.54 (0.34, 0.62)	0.16 (0.05, 0.42)	0.27 (0.20, 0.47)
**B cells**						
CD19+	cells/mm^3^	157 (128, 244)	128 (102, 211)	49 (20, 87)	122 (94, 176)	51 (29, 119)
CD27-IgD+	% CD19+	68.85 (52.03, 74.47)	68.89 (57.94, 77.24)	49.52 (10.73, 80.43)	90.89 (82.94, 93.31)	84.03 (70.93, 90.82)
CD27-IgD-	% CD19+	2.80 (2.18, 4.38)	3.90 (2.24, 6.42)	6.13 (2.64, 23.40)	2.40 (1.67, 2.58)	2.65 (1.72, 4.22)
CD27 **+** IgD+	% CD19+	15.01 (10.11, 23.19)	10.53 (4.42, 13.73)	5.67 (2.43, 8.57)	3.57 (2.21, 7.20)	4.92 (1.32, 7.92)
CD27 **+** IgD-	% CD19+	14.39 (7.08, 20.05)	13.93 (7.26, 24.33)	7.77 (2.43, 19.38)	2.55 (0.94, 3.91)	2.97 (0.36, 6.66)
**T cells**						
CD3+	cells/mm^3^	1363 (1076, 1787)	1045 (883, 1456)	2756 (1472, 4183)	831 (658, 1075)	579 (407, 837)
CD4+	cells/mm^3^	835 (571, 1214)	789 (573, 967)	848 (609, 1227)	382 (302, 565)	308 (244, 565)
CD8+	cells/mm^3^	477 (358, 604)	270 (159, 383)	1621 (710, 2682)	293 (196, 362)	213 (141, 266)
CD4+/CD8+ ratio		1.89 (1.45, 2.42)	2.77 (2.31, 4.08)	0.60 (0.42, 1.06)	1.26 (0.67, 2.65)	1.89 (1.17, 2.61)
***CD8+ T cells***						
CD45RA + CCR7+	% CD8+	19.05 (11.11, 34.59)	28.47 (13.32, 43.67)	1.52 (0.56, 2.75)	4.91 (1.53, 53.19)	35.56 (10.29, 53.19)
CD45RA-CCR7+	% CD8+	0.83 (0.46, 1.90)	4.60 (1.96, 7.97)	0.53 (0.21, 0.82)	0.82 (0.30, 1.20)	1.37 (0.85, 1.96)
CD45RA-CCR7-	% CD8+	31.28 (24.26, 36.96)	43.13 (32.60, 57.14)	36.82 (16.56, 57.25)	39.24 (30.69, 59.20)	29.14 (17.47, 42.55)
CD45RA + CCR7-	% CD8+	41.48 (32.61, 52.32)	22.30 (16.87, 27.10)	40.75 (13.96, 59.90)	48.28 (30.47, 63.41)	29.25 (18.76, 39.37)
CD8 + CD57+	% CD8+	17.52 (9.42, 30.97)	7.38 (4.41, 15.97)	27.36 (19.36, 47.30)	39.61 (33.78, 51.16)	13.51 (8.35, 23.44)
***Activated T cells***						
CD3 + HLA-DR+	% CD3+	12.04 (7.62, 16.95)	9.87 (7.11, 12.83)	52.00 (32.71, 59.56)	46.56 (40.76, 57.25)	13.65 (6.19, 20.33)
CD4 + HLA-DR+	% CD4+	5.88 (4.94, 9.17)	6.75 (4.95, 9.13)	48.30 (26.32, 56.25)	43.87 (30.88, 49.95)	11.54 (6.52, 18.33)
CD8 + HLA-DR+	% CD8+	25.48 (14.86, 31.93)	19.73 (14.18, 26.44)	53.48 (37.05, 65.52)	62.14 (47.01, 76.39)	15.12 (10.65, 28.64)
**Regulatory T cells**	cells/mm^3^	54 (33, 76)	41 (24, 82)	18 (13, 22)	19 (12, 24)	15 (8, 18)
**NK cells**	cells/mm^3^	143 (100, 214)	174 (121, 230)	268 (146, 388)	75 (55, 103)	41 (26, 70)
**Dendritic cells**						
Myeloid	/ml	11940 (8751, 17805)	11422 (6616, 13457)	9122 (5662, 29989)	7795 (4086, 10548)	5420 (4107, 9683)
Plasmacytoid	/ml	7217 (4801, 11084)	6152 (3599, 9836)	2771 (1766, 8490)	3073 (1351, 3618)	2766 (2194, 4255)
**Gammadelta cells**	cells/mm^3^	44 (20, 74)	27 (11, 54)	396 (178, 579)	19 (6, 92)	16 (9, 26)
**Gammadelta 2**	% GD	62.96 (40.85, 73.68)	78.71 (75.00, 89.39)	13.40 (8.77, 30.62)	6.59 (4.09, 25.61)	25.00 (12.41, 55.96)

Results are expressed as medians (interquartile range values).

### PHID complications according to clusters and T-cell activation

Interestingly, the proportion of patients with PHID complications (information not used in the previous analysis) varied significantly between the clusters: 15%, 33%, 75%, 88% and 63% in clusters 1 to 5 respectively (Figure 3, Table 4). As shown by the dendrogram (Figure 2), clusters 3, 4 and 5 were closer to each other than clusters 1 and 2, in which the rate of complications was the lowest. Among the 8 patients in cluster 4, 7 (88%) presented with a splenomegaly (14 splenomegaly recorded in the cohort) and 4 (50%) with a granulomatous disease (9 granulomatous diseases recorded in the cohort). Patients in cluster 5, characterized by a severe loss of switched memory B cells, also presented a high percentage of complications (63%) (Figure 3, Table 4). In Figure 3, the highest percentages of complications (from clusters 3 and 4) are in the upper-left corner, and the lowest (from cluster 1) on the upper-right. Hence, visually, complication rates increased in a clockwise manner.

**Table 4:** Type of PHID and PHID complications in 5 clusters of 79 PHID patients, ALTADIH Cohort, 2007-2010

	**Cluster 1**		**Cluster 2**		**Cluster 3**		**Cluster 4**		**Cluster 5**	
	**n = 33**		**n = 18**		**n = 4**		**n = 8**		**n = 16**	
	**n**	**%**	**n**	**%**	**n**	**%**	**n**	**%**	**n**	**%**
**Type of PHID**										
CVID	23	(70)	8	(44)	3	(75)	8	(100)	13	(82)
IgG subclass deficiency	10	(30)	10	(56)	0	(0)	0	(0)	1	(6)
Good’s syndrome	0	(0)	0	(0)	1	(25)	0	(0)	2	(12)
**PHID complications (at least one of the following)**	**5**	**(15)**	**6**	**(33)**	**3**	**(75)**	**7**	**(88)**	**10**	**(63)**
Lymphoid hyperplasia	2	(6)	5	(28)	1	(25)	3	(38)	5	(31)
Splenomegaly	1	(3)	2	(11)	1	(25)	7	(88)	3	(19)
Autoimmune manifestations	3	(9)	1	(5)	1	(25)	2	(25)	7	(44)
Granulomatous disease	0	(0)	1	(5)	2	(50)	4	(50)	2	(13)
Villous atrophy	0	(0)	2	(11)	0	(0)	0	(0)	3	(19)
Chronic inflammatory intestinal disease	0	(0)	2	(11)	1	(25)	1	(13)	0	(0)

To quantify the independent effects of immunological markers, we then performed a supervised analysis based on a logistic regression model. As the activated HLA-DR + CD3+, CD4+ and CD8+ T-cells were correlated positively in the PCA, CD8+ HLA-DR + was chosen for the ascendant multivariable analysis (as it was the most significantly associated with PHID complications in univariable analysis (Table 5). The final model contained the following three markers. Increased CD8+ HLA-DR + T-cells were strongly associated with PHID complications (odds ratio per one standard deviation = 2.55; confidence interval [1.33; 4.89]; p-value = 0.0047). Both decreased plasmacytoid dendritic cells (odds ratio per one standard deviation = 0.49; confidence interval [0.25; 0.96]; p value = 0.0363) and decreased switched memory B cells (odds ratio per one standard deviation = 0.51; confidence interval [0.28; 0.94]; p value = 0.0312) were also associated with PHID complications.

**Table 5:** Immunological markers association with CVID complications

	**Unit**	**Odds ratio**	**95% confidence interval**	**p-value**
**B cells**				
CD19+	cells/mm^3^	0.65	[0.36; 1.15]	0.1373
CD27-IgD+	% CD19+	1.49	[0.89; 2.48]	0.1310
CD27-IgD-	% CD19+	0.54	[0.24; 1.23]	0.1401
CD27 **+** IgD+	% CD19+	0.62	[0.34; 1.12]	0.1124
CD27 **+** IgD-	% CD19+	0.93	[0.29; 0.86]	0.0119
**T cells**				
CD3+	cells/mm^3^	0.68	[0.40; 1.17]	0,1646
CD4+	cells/mm^3^	0.45	[0.26; 0.78 ]	0,0046
CD8+	cells/mm^3^	1.04	[0.66; 1.62 ]	0.8818
CD4+/CD8+ ratio		0.99	[0.63; 1.56 ]	0.9649
***CD8+ T cells***				
CD45RA + CCR7+	% CD8+	0.69	[0.43; 1.13]	0.1385
CD45RA-CCR7+	% CD8+	0.75	[0.46; 1.25]	0.2710
CD45RA-CCR7-	% CD8+	1.38	[0.86; 2.20]	0.1800
CD45RA + CCR7-	% CD8+	0.77	[0.48; 1.23]	0.2666
CD8 + CD57+	% CD8+	1.30	[0.83; 2.06]	0.2567
***Activated T cells***				
CD3 + HLA-DR+	% CD3+	2.99	[1.56; 5.73]	0.0010
CD4 + HLA-DR+	% CD4+	2.70	[1.40; 5.23]	0.0032
CD8 + HLA-DR+	% CD8+	2.75	[1.54; 4.90]	0.0006
**Regulatory T cells**	cells/mm^3^	0.41	[0.22; 0.78]	0.0065
**Natural Killer cells**	cells/mm^3^	0.75	[0.46; 1.21]	0.2369
**Dendritic cells**				
Myeloid	/ml	0.65	[0.36; 1.17]	0.1532
Plasmacytoid	/ml	0.37	[0.18; 0.73]	0.0046
**Gammadelta cells**	cells/mm^3^	1.30	[0.79; 2.13]	0.3028
Gammadelta 2 cells	% GD	0.52	[0.32; 0.85]	0.0087

Univariable logistic regression analysis (Odds ratio per 1 standard deviation).

ALTADIH Cohort, 2007-2010.

### Classification of CVID patients according to EUROclass and CD8+ HLA-DR + marker

In symptomatic PHID, the only disease for which a consensual classification exists is CVID. Among the 57 CVID patients in the ALTADIH cohort, EUROclass distinguishes 1 patient with equal or less than 1% of B cells of lymphocytes (B- group) from the 56 patients with a higher percentage (B + group). B + patients were divided into 15 patients with severe deficiency of class-switched memory B cells (≤ 2% of B cells, smB- group) and 40 patients with more than 2% of class-switched memory B cells (smB + group). The smB status of one patient could not be evaluated. EUROclass also discriminates between patients according to the expansion of CD21^low^ B cells: above or below 10% within B cells (CD21^low^ versus CD21^norm^). In our study, among 14 PHID patients with splenomegaly, 11 (79%) were classified in the CD21^low^ group.

As we found a strong association between PHID complications and increased CD8+ HLA-DR + T-cells (expressed as percentages of CD8+ lymphocytes) and because CD8+ HLA-DR + has been involved in CVID [12], we examined whether the inclusion of CD8+ HLA-DR + in addition to EUROclass criteria, would improve the prediction of CVID complications (Table 6). Among 19 patients with CVID complications and classified with less than 50% risk using EUROclass, 10 were reclassified with a risk greater than 50% when adding the CD8+ HLA-DR + activation marker. Moreover, the addition of CD8+ HLA-DR + to the model increased its predictive value (c statistic 0.57 without CD8+ HLA-DR + versus 0.77 with CD8+ HLA-DR+, a value close to 1 indicating a good prediction). Specifically, adding CD8 + HLA-DR + to EUROclass model leads to a substantial increase in sensitivity (from 24% to 60%) and some loss of specificity (from 90% to 80%) (Additional file 7: Tables S3 and Additional file 8: Table S4).

**Table 6:** Reclassification of 55 CVID patients smB+/-, separated according to experience of complications

**Model with EUROclass**	**Model with EUROclass and CD8+ HLA-DR+**		**Total**
**Predicted probabilities (%)**	**< 50%**	**≥ 50%**	
**Patients who experienced CVID complications**			
< 50%	9		19
≥ 50%	1	5	6
Total	10	15	25
**Patients who didn’t experience CVID complications**			
< 50%	22	5	27
≥ 50%	2	1	3
Total	24	6	30

ALTADIH Cohort, 2007-2010.

The agreements between the two models for classifying the patients are depicted in gray. Adding CD8 + HLA-DR + to EUROclass model leads to better determine CVID complications for 10 patients who experienced CVID complications (upward movement in category for patients who experienced CVID complications) and to better determine the absence of CVID complications for 2 patients who did not experience CVID complications (downward movement in category for patients who did not experience CVID complications). In parallel, it leads to worsened classification for 1 patient who experienced CVID complications (downward movement in category for patients who did experience CVID complications) and 5 patients who did not experience CVID complications (upward movement in category for patients who did not experience CVID complications).

The positive net reclassification improvement (0.26) revealed that CD8+ HLA-DR + offers improved prediction of CVID complications.

## Discussion

In the present study, we focused on three PHID in adults with symptomatic implications: CVID, IgG SD and Good’s syndrome (referred in the 2011 update of Primary Immunodeficiency by the International Union of Immunological Societies [1]). We used unsupervised and supervised statistical methods on a cohort of 81 PHID patients in an attempt to select relevant markers that may be useful for diagnosis and prognosis of these patients.

Our rationale to pool patients diagnosed with CVID, IgG SubClass Immuno deficient and Good`s patients, and thus, to include in the ALTADIH cohort the active file of PHID patients with symptoms in Aquitaine, South-Western France, was based on the following arguments: 1) Because of its complexity, distinguishing patients with symptomatic PHID can be challenging for the diagnosis and the treatment. That is why we did not focus only on CVID patients, and we wanted to address the symptomatic PHID population seen in clinical routine practice by physicians, including IgG SubClass Immuno deficient patients as well as Good's patients; 2) Literature on IgG subclass deficiency is poor and immunological characteristics of these patients have not been explored as they have been in CVID. Indeed, IgG subclass deficiency may progress to have typical CVID [3]. Thus, the study of both CVID and IgG subclass deficiency in a combined analysis was a novel aspect of our work; 3) Good syndrome (thymoma with immunodeficiency) was listed as a predominantly antibody deficiency in the 2009 update of Primary Immunodeficiency by the International Union of Immunological Societies [1]. Based on our view to include eligible patients with symptomatic PHID seen in routine clinical practice and this pivotal article, we do not have any arguments to exclude it from eligibility criteria.

First, PCA gave some insight to the association between the 23 immunological markers measured. An increase in the proportion of HLA-DR + T-cell as an indication for activation, positively correlated to an increase in CD45RA + CCR7- terminal effector CD8+ T-cells and to the CD8 + CD57+ immunosenescence marker, defined one group of markers. In parallel, a defect in B-cell differentiation (increased CD19 + CD27-IgD + naïve B cells and by symmetry, decreased CD19 + CD27 + IgD- switched memory B cells) might identify another distinct pathway. The PCA gave the opportunity to explore and to observe graphically the links between immunological markers and we made the choice to present the plane spanned by the two first principal components because they explained the most the data variability (43%). Next, we performed a hierarchical cluster analysis on the basis of the cell profile under investigation. As it was more relevant biologically, cluster analysis was performed with immunological markers rather than with principal components. Thus, the variance left on other principal components was not an obstacle for the cluster analysis.

The cluster analysis separated five major groups in our PHID population with distinct rates of complications. Particularly, patients in cluster 5 (13 with CVID, 1 with IgG SD and 2 with Good’s syndrome), 63% of whom suffering PHID complications, were characterized by a severe loss of CD19 + CD27 + IgD- switched memory B-cells whereas patients in clusters 3 and 4, mainly with CVID (75% and 88% of whom having PHID complications, respectively) were characterized by increased HLA-DR + activation of T-cells. Regarding available data for activated T cells or B cell subsets for 12 normal healthy donors (Additional file 4: Table S2) and data from PHID patients in Table 3, activated T cells were higher in PHID patients from clusters 3 and 4, and switched memory B cells were particularly lower in PHID patients from clusters 4 and 5. Furthermore, the strong association we found between PHID complications and increased CD8+ HLA-DR + T-cells reinforced the importance of the link between T-cell activation and clinical complications. More importantly, in a substudy with CVID patients, the classification that added CD8+ HLA-DR + to EUROclass was superior to the EUROclass model in predicting CVID complications. The advantage of adding CD8+ HLA-DR + to EUROclass resulted in a substantial increase of sensitivity and some loss of specificity to determine CVID complications. As a consensus does not exist for the cut-off of probability, we explored a threshold of 50% (Additional file 7: Tables S3, Additional file 8: Table S4).

Our results on switched memory B-cell defect, defining PHID cluster 5 in particular, are consistent with previous findings on B-cell phenotype in PHID [4,5,9,10]. Our study also highlights the importance of considering T-cell activation [12,13] in PHID (clusters 3 and 4 with increased T-cell activation with the highest percentage of complications, comprising mainly CVID patients and the three patients with Good’s syndrome identified in our cohort as PHID types). Moreover, the occurrence of two complications considered in addition to those defined by H Chapel et al. [6] (ie: splenomegaly and granulomatous disease) were found with the highest frequency in the clusters 3 and 4 with increased T-cell activation, suggesting that the HLA-DR + activation marker could help to discriminate them. However, this cross-sectional analysis of data from the ALTADIH cohort does not allow us to determine whether increased T-cell activation is a causal factor of PHID progression or if it is induced by PHID complication. Only longitudinal analyses of activation markers would help in deciphering the role of T-cell activation, determining whether or not patients with normal phenotype will progress to an abnormal phenotype and will develop complications. Adding T-cell parameters for discriminating PHID patients is important to better dissect these heterogeneous syndromes. This has been previously suggested in CVID [31] by several authors especially the Rome group [11] (Giovannetti et al.) as well as the Czech group [32] (Vlková et al.) and the French DEFI group [7] (Mouillot et al.). These authors suggested CD4 naïve T cell percentage as additional marker in CVID. Markers of CD4+ T-cell differentiation were not performed in our study. At the time of research protocol writing, set up in 2005, the importance of CD4+ T-cell differentiation has not been reported in PHID literature as it would be few years later. Thus, markers of CD4+ T-cell differentiation have not been planned in the ALTADIH protocol and the prospective design of the study did not allow for this B-cell marker to be assessed afterwards. This is a clear limitation of the study. However, previous authors also highlighted that a decrease of naive CD4 cells was also associated to T-cell activation and it would not be unreasonable to consider T-cell activation as an indirect marker of decreased naive CD4 cells in our study. In addition, the signification of the memory status in the CD4+ compartment has been a subject of debates until recently as exemplified by Su LF, et al. [33].

## Conclusion

To date, PHID diseases have only been examined through the analysis of separated compartments of the immune system. On this ground, our approach of combining a wide set of immunological markers using appropriate statistical methods for the high-dimensional is a novel aspect in PHID. The meaning of the identified clusters would need to be validated with a larger number of patients, for example the French national prospective Cohort DEFI [7]. It would be the opportunity to include markers of CD4+ T-cell differentiation in the analyses and consider potential other markers, as mucosal associated invariant T cells [34,35], recently reported in CVID [36].

In conclusion, by combining a wide set of immunological markers, this study reveals that T-cell activation could be of additional value to discriminate patients with specific PHID features. Such information might have implications for diagnosis and clinical management. A systematic recording of the HLA-DR + phenotype in every patient with PHID seems relevant.

## Abbreviations

CVID: Common Variable ImmunoDeficiency; GD: GammaDelta T cells; Ig: Immunoglobulin; IgG SD: IgG SubClass ImmunoDeficiency; IQR: InterQuartile Range; mab: Monoclonal antibody; mDC: Myeloid dendritic cells; NK: Natural Killer cells; OR: Odds Ratio; PCA: Principal Component Analysis; PC: Principal Component; PC1: First Principal Component; PC2: Second Principal Component; pDC: Plasmacytoid Dendritic Cells; PHID: Primary Humoral Immunodeficiency Diseases; SD: Standard Deviation; smB: Switched memory B cells; T reg: Regulatory T cells.

## Competing interests

The authors declare that they have no competing interests.

## Authors’ contributions

DA, XD, A-LF, FL, PR, and J-FV enrolled PHID patients in the cohort and provided clinical data. J-FM performed the flowcytometric analysis. M-QP was involved in data collection. M-QP and RT participated in the study design and performed the statistical analysis of the data. M-QP wrote the manuscript. J-FM, RT, LW and J-FV critically read the manuscript. All authors read and approved the final manuscript.

## ALTADIH study group members

Scientific committee: Dr F. Lifermann, Pr J-F. Moreau, R. Thiébaut, Pr J-F. Viallard Participating Hospital Departments (participating physicians): Bordeaux University Hospital (Pr J-F. Viallard); Agen Hospital (Dr P. Rispal); Dax Hospital (Dr F. Lifermann); Limoges Hospital (Dr A-L. Faucher); Pau Hospital (Dr X. Delbrel); Toulouse Hospital (Pr D. Adoue); Bordeaux Immunology Department: Pr P. Blanco, J-C. Carron, K. Eschechel, M. Garcie, Pr J-F. Moreau, Dr I. Pellegrin

Methodology: R. Thiébaut, M-Q. Picat, L. Wittkop

Data collection: D. Jacquemart, M-Q. Picat

Computing and data management: G. Dupouy, C. Maldonado, I. Perrot

Statistical analysis: M-Q. Picat, R. Thiébaut

## Supplementary Material

Additional file 1: Table S1 References of the panels of monoclonal antibodies used for identifying cells by flow cytometry using a FC500 flow cytometer from Beckman-Coulter. Click here for file

Additional file 2: Figure S1 Gating strategies for for flow cytometric assessment of activated T-lymphocytes and other major lymphocyte sub-populations in the blood on a CVID patient. Click here for file

Additional file 3: Figure S2 Immunological markers of 12 healthy donors, plotted on the first two principal components. Click here for file

Additional file 4: Table S2 Immunological marker values of normal patients. Click here for file

Additional file 5: Figure S3 Thirty-eight immunological markers involved in PHID on the two first principal components. ALTADIH Cohort, 2007-2010. Click here for file

Additional file 6: Figure S4 Classification of 55/57 CVID patients by hierarchical cluster analysis, according to 23 immunological markers involved in the disease. ALTADIH Cohort, 2007-2010. Click here for file

Additional file 7: Table S3 Sensibility [95% confidence interval] and specificity [95% confidence interval] of the model EUROclass to determine CVID complications with a probability ≥ 50% or < 50%. Click here for file

Additional file 8: Table S4 Sensibility [95% confidence interval] and specificity [95% confidence interval] of the model EUROclass and CD8+HLA-DR+ to determine CVID complications with a probability ≥ 50% or < 50%. Click here for file
