# Supplementary material for: T-cell activation discriminates subclasses of symptomatic primary humoral immunodeficiency diseases in adults
Source: BMC Immunol. 2014 Mar 12;15:13. doi: 10.1186/1471-2172-15-13 (PMC4008268; doi:10.1186/1471-2172-15-13)
Supplement: Additional file 1: Table S1 — References of the panels of monoclonal antibodies used for identifying cells by flow cytometry using a FC500 flow cytometer from Beckman-Coulter. [file 1471-2172-15-13-S1.doc]

**Additional file 1:Table S1. References of the panels of monoclonal antibodies used for identifying cells by flow cytometry using a FC500 flow cytometer from Beckman-Coulter.**

| **Cells labelling*** | **Monoclonal antibodies** | **References** |
| --- | --- | --- |
| **B-cell differentiation** | anti-CD19 | A07766 |
|  | anti-CD27 | 6607107 |
|  | anti-CD21 | PN IMU473U |
|  | anti-sIgD | 736000 |
| **T-cell differentiation** | anti-CD8 | A07756 |
|  | anti-CD45RA | PN IM 271U |
|  | anti-CCR7 from R&D (Minneapolis, MN, USA) | FAB197A |
| **HLA-DR T-lymphocyte activation markers** | TetraChrome  (anti-CD45, -3, -4 and -8 | 6607013 |
|  | anti-HLA-DR | PNA40579 |
| **CD25 T-lymphocyte activation marker** | anti-CD3, -4 and -8 | 6607013 |
|  | anti-CD25 | IM2646 |
| **CD28 T-lymphocyte activation marker** | anti-CD3, -4 and -8 | 6607013 |
|  | anti-CD28 | 6607108 |
| **CD38 T-lymphocyte activation marker** | anti-CD3, -4 and -8 | 6607013 |
|  | anti-CD38 | A07780 |
| **Regulatory T-cells** | anti-CD4 | A07750 |
|  | anti-CD25 | IM2646 |
|  | anti-CD127 | PN IM1980U |
| **NK/B cells** | anti-CD45, -56, -19 and -3 | 6607073 |
|  | anti-CD16 | A07766 |
| **Myeloid dendritic cells** | Beckman Coulter kit | A23413 |
| **Plasmacytoid dendritic cells** | Beckman Coulter kit | A23416 |
| **T cells expressing the gamma/delta TCR** | anti-Vdelta2 | PN IM1464 |
|  | anti-pan-delta | PN IM1418U |

**T and B-cell subpopulation counts were obtained using the flow count beads kit from Beckman-Coulter Flow Count (ref: 7547053) following a lyse (Versalyse ref: A09777) and no wash procedure according to the manufacturer’s recommendations.*
