# Supplementary figures and images for: T-cell activation discriminates subclasses of symptomatic primary humoral immunodeficiency diseases in adults
Source: BMC Immunol. 2014 Mar 12;15:13. doi: 10.1186/1471-2172-15-13 (PMC4008268; doi:10.1186/1471-2172-15-13)

# A

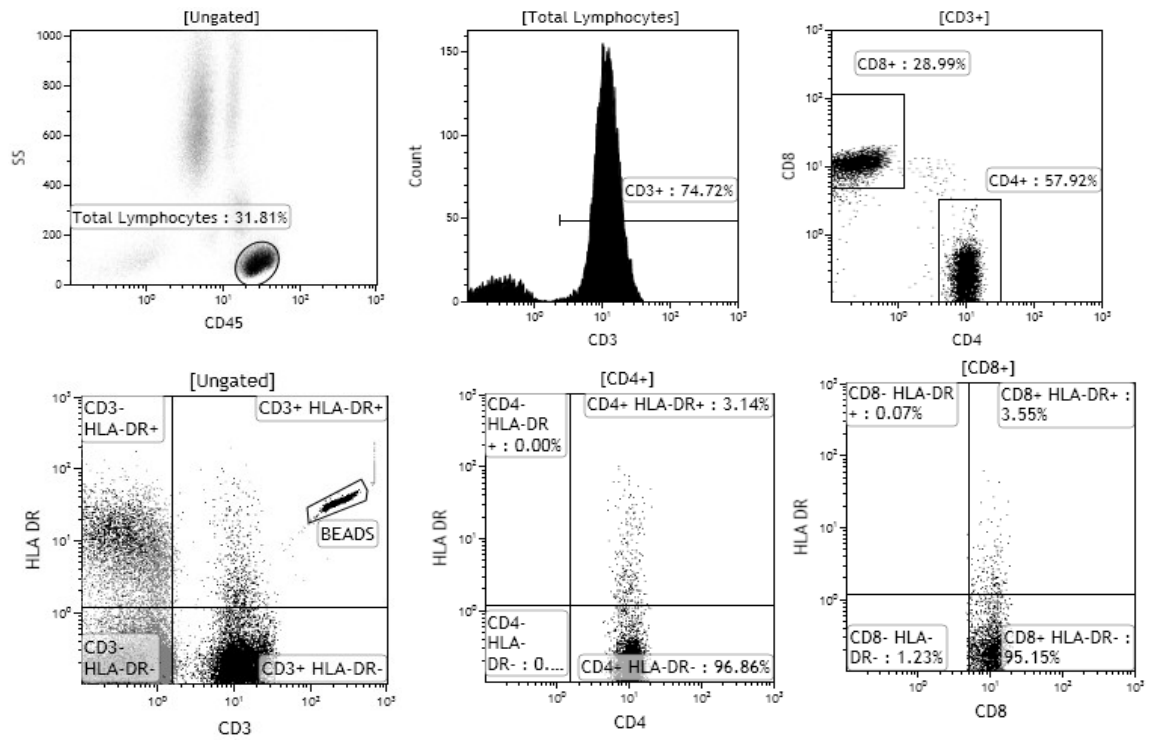

# B

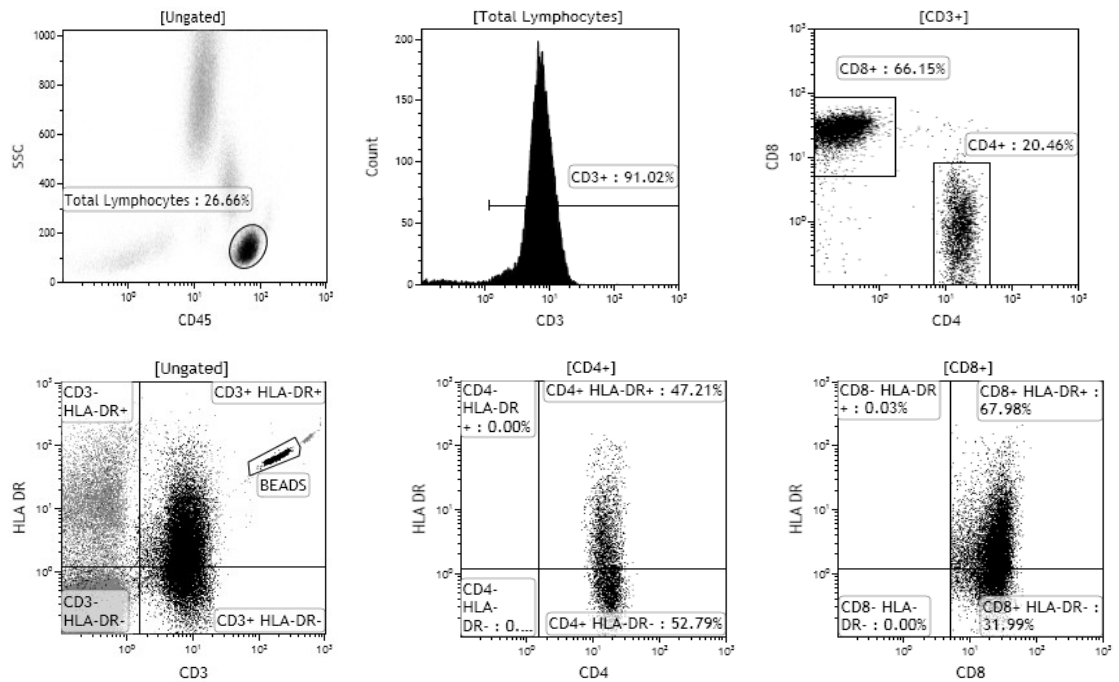

# C

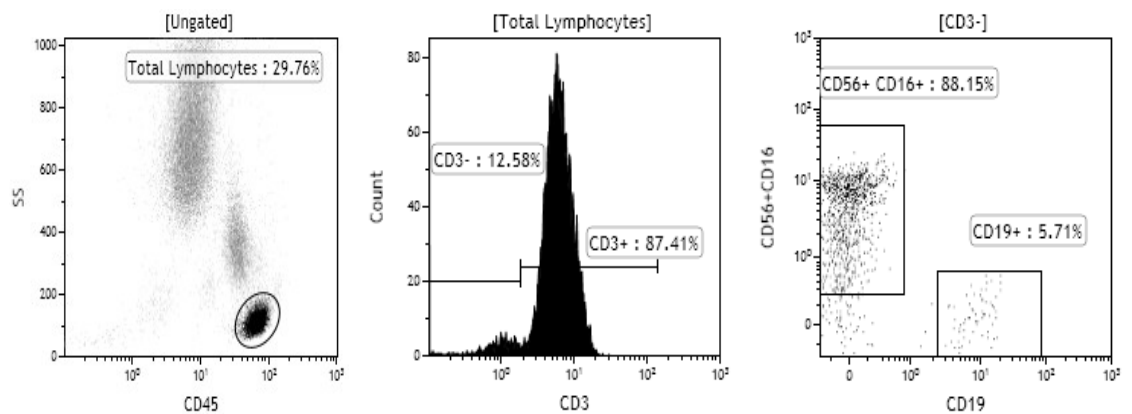

Supplement: Additional file 2: Figure S1 — Gating strategies for for flow cytometric assessment of activated T-lymphocytes and other major lymphocyte sub-populations in the blood on a CVID patient. [file 1471-2172-15-13-S2.pdf]

**
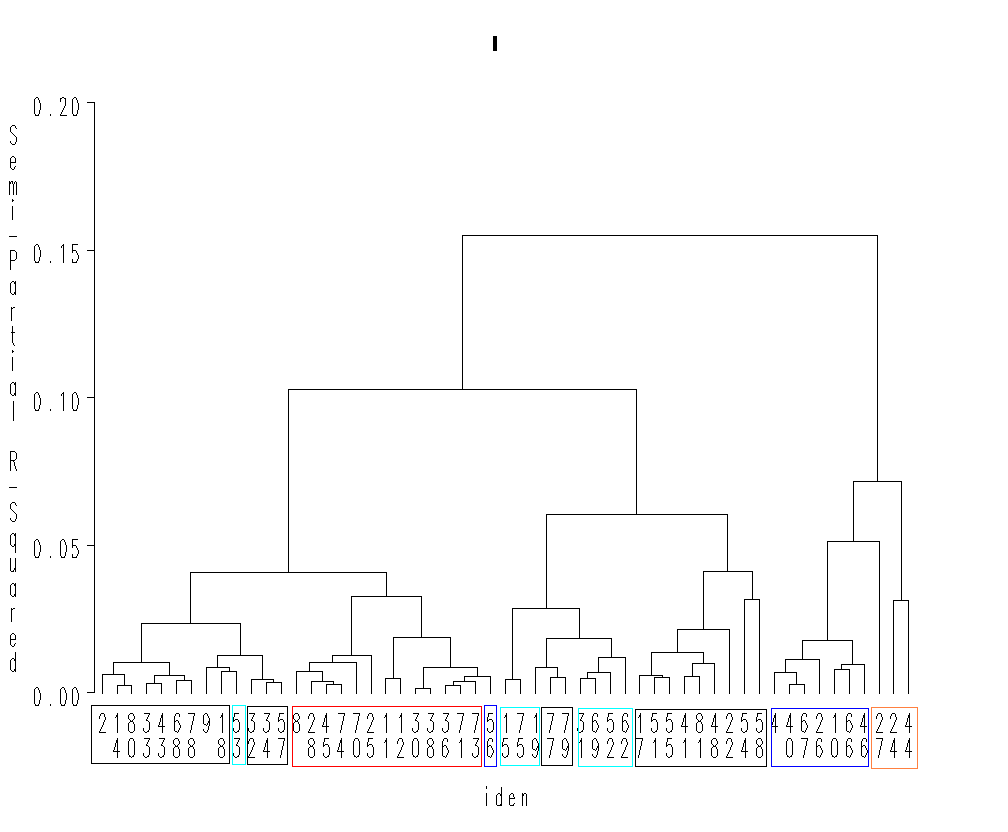
**

Supplement: Additional file 6: Figure S4 — Classification of 55/57 CVID patients by hierarchical cluster analysis, according to 23 immunological markers involved in the disease. ALTADIH Cohort, 2007-2010. [file 1471-2172-15-13-S6.doc]
