# Supplementary material for: T-cell activation discriminates subclasses of symptomatic primary humoral immunodeficiency diseases in adults
Source: BMC Immunol. 2014 Mar 12;15:13. doi: 10.1186/1471-2172-15-13 (PMC4008268; doi:10.1186/1471-2172-15-13)
Supplement: Additional file 4: Table S2 — Immunological marker values of normal patients. [file 1471-2172-15-13-S4.doc]

**Additional file 4: Table S2. Immunological marker values of normal patients.**

|  |  | **Unit** | **Records** | **Median** | **IQR** |
| --- | --- | --- | --- | --- | --- |
| **Total circulating lymphocytes (TCL)** | | cells/mm^3^ | 101* | 2100 | (1600, 2400) |
| **B cells** | CD19+ | cells/mm^3^ | 101* | 300 | (200, 400) |
|  |  | % TCL | 101* | 32 | (28, 39) |
| Naïve | CD27-IgD+ | % CD19+ | 12^†^ | 67.45 | (55.49, 74.58) |
| Switched | CD27-IgD- | % CD19+ | 12^†^ | 2.75 | (1.82, 6.48) |
| Marginal zone | CD27**+**IgD+ | % CD19+ | 12^†^ | 9.25 | (6.07, 15.45) |
| Switched memory | CD27**+**IgD- | % CD19+ | 12^†^ | 16.04 | (11.88, 20.01) |
| **T cells** | CD3+ | cells/mm^3^ | 101* | 1400 | (1100, 1700) |
|  | CD4+ | cells/mm^3^ | 101* | 800 | (700, 1100) |
|  | CD8+ | cells/mm^3^ | 101* | 700 | (500, 900) |
|  | CD4+/CD8+ ratio |  | 101* | 1.2 | (1.0, 1.5) |
| Naïve | CD45RA+CCR7+ | % CD8+ | 8^†^ | 55.64 | (44.57, 62.19) |
| Central memory | CD45RA-CCR7+ | % CD8+ | 8^†^ | 0.86 | (0.57, 1.32) |
| Effector memory | CD45RA-CCR7- | % CD8+ | 8^†^ | 23.13 | (17.89, 32.09) |
| Terminal  effector | CD45RA+CCR7- | % CD8+ | 8^†^ | 31.87 | (13.97, 24,03) |
| Immunosenescent | CD8+CD57+ | % CD8+ | 8^†^ | 1.11 | (0.92, 4.20) |
| ***Activated T cells*** |  |  |  |  |  |
| HLA-DR | CD3+HLA-DR+ | % CD3+ | 12^†^ | 6,36 | (5,04, 8.29) |
|  | CD4+HLA-DR+ | % CD4+ | 12^†^ | 6,63 | (4.25, 7.60) |
|  | CD8+HLA-DR+ | % CD8+ | 12^†^ | 14,68 | (10.12, 19.68) |
| **Regulatory T cells** | CD4+CD25+CD127- | cells/mm^3^ | 12^†^ | 54 | (39, 79) |
| **Natural Killer cells** | CD3-CD16+CD56+ | cells/mm^3^ |  | 225 | (158, 294) |
| **Dendritic cells** |  |  |  |  |  |
| Myeloid | mDC | /ml | 12^†^ | 14547 | (8544, 20648) |
| Plasmacytoid | pDC | /ml | 12^†^ | 7233 | (5551, 9617) |
| **Gammadelta cells** | | cells/mm^3^ | 8^†^ | 52 | (43, 94) |
| Gammadelta 2 cells | | % GD | 8^†^ | 1.90 | (1.43, 4.13) |

*Data from 101 normal adults (45 male, 56 female) aged 18 to 70 years, with a median age of 40 years. [Erkeller-Yuksel FM](http://www.ncbi.nlm.nih.gov/pubmed?term=Erkeller-Yuksel FM%5BAuthor%5D&cauthor=true&cauthor_uid=1735817) et al. [J Pediatr](http://www.ncbi.nlm.nih.gov/pubmed/1735817) 1992 Feb;120:216-22.

^†^Data available from 12 normal adults (4 male, 8 female) aged 23 to 56 years, with a median age of 31 years (missing values for some markers).
