# Supplementary material for: T-cell activation discriminates subclasses of symptomatic primary humoral immunodeficiency diseases in adults
Source: BMC Immunol. 2014 Mar 12;15:13. doi: 10.1186/1471-2172-15-13 (PMC4008268; doi:10.1186/1471-2172-15-13)
Supplement: Additional file 7: Table S3 — Sensibility [95% confidence interval] and specificity [95% confidence interval] of the model EUROclass to determine CVID complications with a probability ≥ 50% or < 50%. [file 1471-2172-15-13-S7.doc]

**Additional file 7: Table S3. Sensibility [95% confidence interval] and specificity [95% confidence interval] of the model EUROclass to determine CVID complications with a probability ≥ 50% or < 50%.**

| Probability of CVID complications predicted by model with EUROclass | Complication = yes | Complication = no | TOTAL |
| --- | --- | --- | --- |
| ≥ 50% | 6 | 3 | 9 |
| < 50% | 19 | 27 | 46 |
| TOTAL | 25 | 30 | 55 |

Sensitivity = 24.0 % [12.7; 35.3]

Specificity = 90.0 % [82.1; 97.9]
