# Supplementary material for: T-cell activation discriminates subclasses of symptomatic primary humoral immunodeficiency diseases in adults
Source: BMC Immunol. 2014 Mar 12;15:13. doi: 10.1186/1471-2172-15-13 (PMC4008268; doi:10.1186/1471-2172-15-13)
Supplement: Additional file 8: Table S4 — Sensibility [95% confidence interval] and specificity [95% confidence interval] of the model EUROclass and CD8+HLA-DR+ to determine CVID complications with a probability ≥ 50% or < 50%. [file 1471-2172-15-13-S8.doc]

**Additional file 8: Table S4. Sensibility [95% confidence interval] and specificity [95% confidence interval] of the model EUROclass and CD8+HLA-DR+ to determine CVID complications with a probability ≥ 50% or < 50%.**

| Probability of CVID complications predicted by model with EUROclass and CD8+ HLA-DR+ | Complication = yes | Complication = no | TOTAL |
| --- | --- | --- | --- |
| ≥ 50% | 15 | 6 | 21 |
| < 50% | 10 | 24 | 34 |
| TOTAL | 25 | 30 | 55 |

Sensitivity = 60.0 % [47.1; 72.9]

Specificity = 80.0 % [67.1; 92.9]
