# Supplementary material for: T-cell activation discriminates subclasses of symptomatic primary humoral immunodeficiency diseases in adults
Source: BMC Immunol. 2014 Mar 12;15:13. doi: 10.1186/1471-2172-15-13 (PMC4008268; doi:10.1186/1471-2172-15-13)
Supplement: Supplementary file 6 [file 1471-2172-15-13-S3.doc]

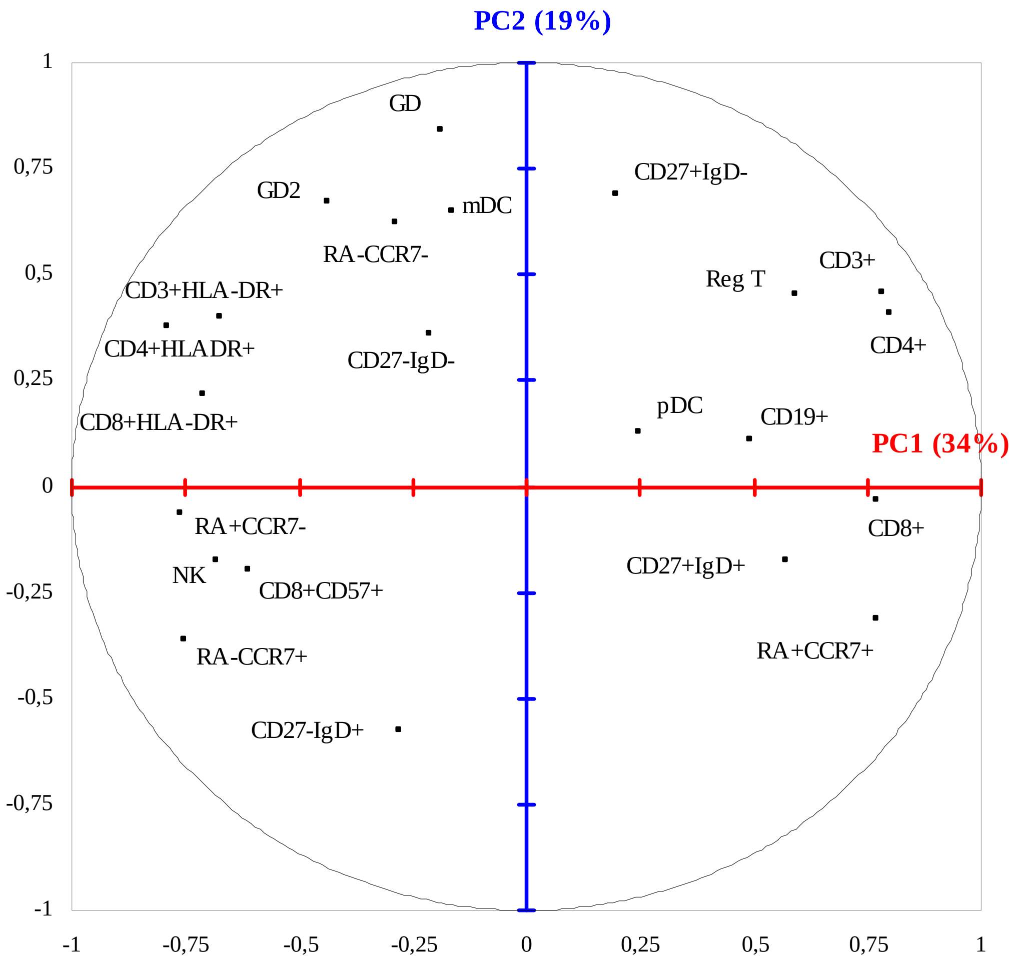
PC2 (19%)
PC1 (34%)
1
0,75
0,5
0,25
0
-0,25
-0,5
-0,75
-1
-1
-0,75
-0,5
-0,25
0
0,25
0,5
0,75
1
GD
GD2
mDC
RA-CCR7-
CD27+IgD-
CD3+HLA-DR+
CD4+HLADR+
CD27-IgD-
Reg T
CD3+
CD4+
CD8+HLA-DR+
pDC
CD19+
RA+CCR7-
NK
CD8+CD57+
CD8+
CD27+IgD+
RA-CCR7+
RA+CCR7+
CD27-IgD+
