# Supplementary material for: T-cell activation discriminates subclasses of symptomatic primary humoral immunodeficiency diseases in adults
Source: BMC Immunol. 2014 Mar 12;15:13. doi: 10.1186/1471-2172-15-13 (PMC4008268; doi:10.1186/1471-2172-15-13)
Supplement: Supplementary file 7 [file 1471-2172-15-13-S5.doc]

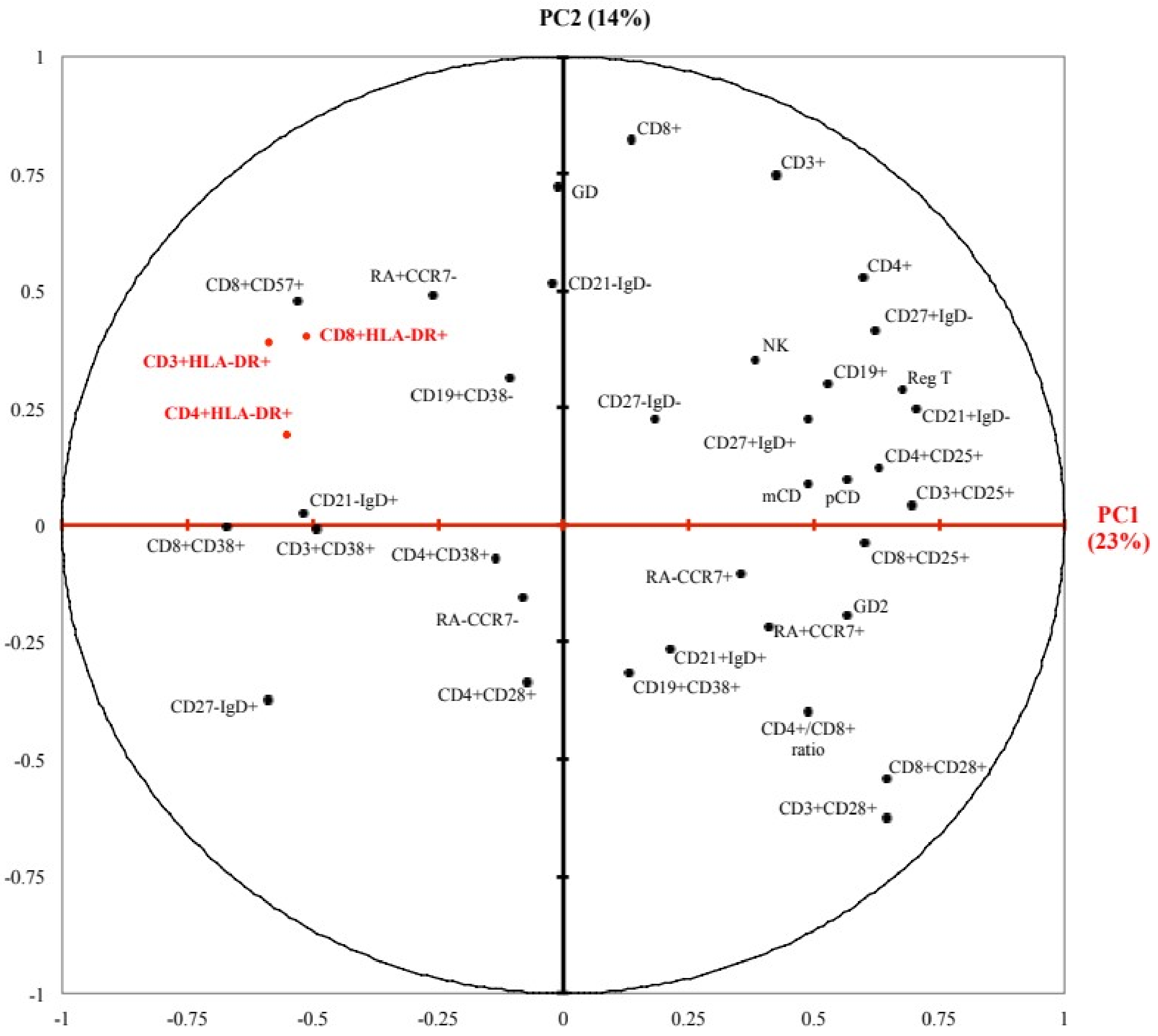
PC2 (14%)
PC1 (23%)
CD8+
CD3+
GD
CD4+
CD8+CD57+
RA+CCR7-
CD21-IgD-
CD27+IgD-
CD8+HLA-DR+
CD3+HLA-DR+
NK
CD19+
Reg T
CD19+CD38-
CD27-IgD-
CD21+IgD-
CD4+HLA-DR+
CD27+IgD+
CD4+CD25+
mCD
pCD
CD3+CD25+
CD21-IgD+
CD8+CD38+
CD3+CD38+
CD4+CD38+
CD8+CD25+
RA-CCR7+
RA-CCR7-
GD2
RA+CCR7+
CD21+IgD+
CD19+CD38+
CD27-IgD+
CD4+CD28+
CD4+/CD8+ ratio
CD8+CD28+
CD3+CD28+
1
0.75
0.5
0.25
0
-0.25
-0.5
-0.75
-1
-1
-0.75
-0.5
-0.25
0
0.25
0.5
0.75
1
